# Supplementary material for: Mapping membrane biophysical nano-environments
Source: Nat Commun. 2024 Nov 7;15:9641. doi: 10.1038/s41467-024-53883-1 (PMC11544141; doi:10.1038/s41467-024-53883-1)
Supplement: Supplementary file 1 — Supplementary Information [file 41467_2024_53883_MOESM1_ESM.pdf]

Mapping membrane biophysical nano-environments

L. Panconi[1,2,3,§], J. Euchner[3,4,5,§], S.A. Tashev [3,4,5], M. Makarova[3,6,7], D-P. Herten[3,4,5], D.M. Owen[1,3,8] & D. J. Nieves[1,3*]

1. Department of Immunology and Immunotherapy, School of Infection, Inflammation and Immunology, College of Medicine and Health, University of Birmingham, Birmingham, UK.
2. School of Physics and Astronomy, College of Engineering and Physical Sciences, University of Birmingham, Birmingham, UK.
3. Centre of Membrane Proteins and Receptors, University of Birmingham, Birmingham, UK.
4. Department of Cardiovascular Sciences, School of Medical Sciences, College of Medicine and Health, University of Birmingham, Birmingham, UK.
5. School of Chemistry, College of Engineering and Physical Sciences, University of Birmingham, Birmingham, UK.
6. School of Biosciences, College of Life and Environmental Science, University of Birmingham, Birmingham, UK.
7. Department of Metabolism and Systems Science, School of Medical Sciences, College of Medicine and Health, University of Birmingham, Birmingham, UK.
8. School of Mathematics, College of Engineering and Physical Sciences, University of Birmingham, Birmingham, UK.

[§] Equal contribution
* Correspondence: d.j.nieves@bham.ac.uk

SUPPLEMENTARY INFORMATION

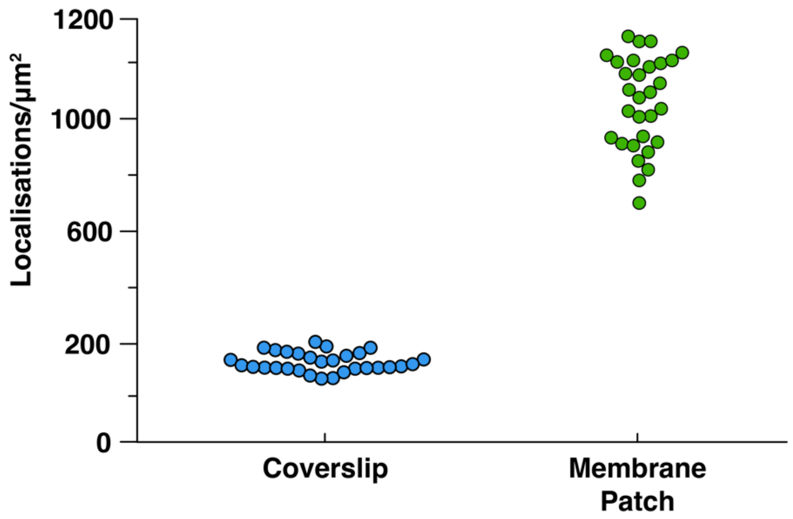

Supplementary Figure 1: **Comparison between detected di-4-ANEPPDHQ binding to the coverglass and DOPC membrane patch.** The number of localisations detected per µm[2] were calculated from DOPC Di-4-ANEPPDHQ PAINT data. 30 ROIs (from two independent experiments) were selected from either the cover glass region (blue) or within the bilayer patch (green).

In addition to the analysis techniques presented here, we have also produced methods for estimating the two key parameters: radius and deviance. Recall that the search radius refers to the maximum distance between any two neighbours of the same cluster. Generally speaking, we can estimate this radius from the maximising value of the Ripley's H-function, as this represents the spatially-averaged distance at which the number of neighbours is maximised (**Supplementary Figure 2a-b**)[1,2]. If the spatial distribution of the point cloud is known to be completely spatially random (CSR), it is advised that the point pattern is first discretised then partitioned into separate categories. This ensures that any underlying clusters are pronounced and allows the parameter estimator to more accurately determine the cluster radius.

The deviance τ can be derived through Gaussian mixture modelling. To do this, we record the continuous marks for each point in turn. We anticipate that this distribution will form two peaks – in the context of SMLM, these represent ordered and disordered GP values respectively (**Supplementary Figure 2c**). Here, we assume that this distribution can be represented as a sum of two Normal curves, given as $f_1 + f_2 = cN(\mu_1, \sigma_1) + (1 - c)N(\mu_2, \sigma_2)$, and use maximum likelihood estimation to ascertain the five unknowns[3]. The deviance defines the maximum difference in density between any two points of the same domains. We therefore aim to maximise deviance without unintentionally absorbing outliers or points from other domains into the clusters. As such, we take the deviance to be the minimum difference between the peaks μ_1, μ_2 and the point of intersection between f_1 and f_2 (**Supplementary Figure 2d**).

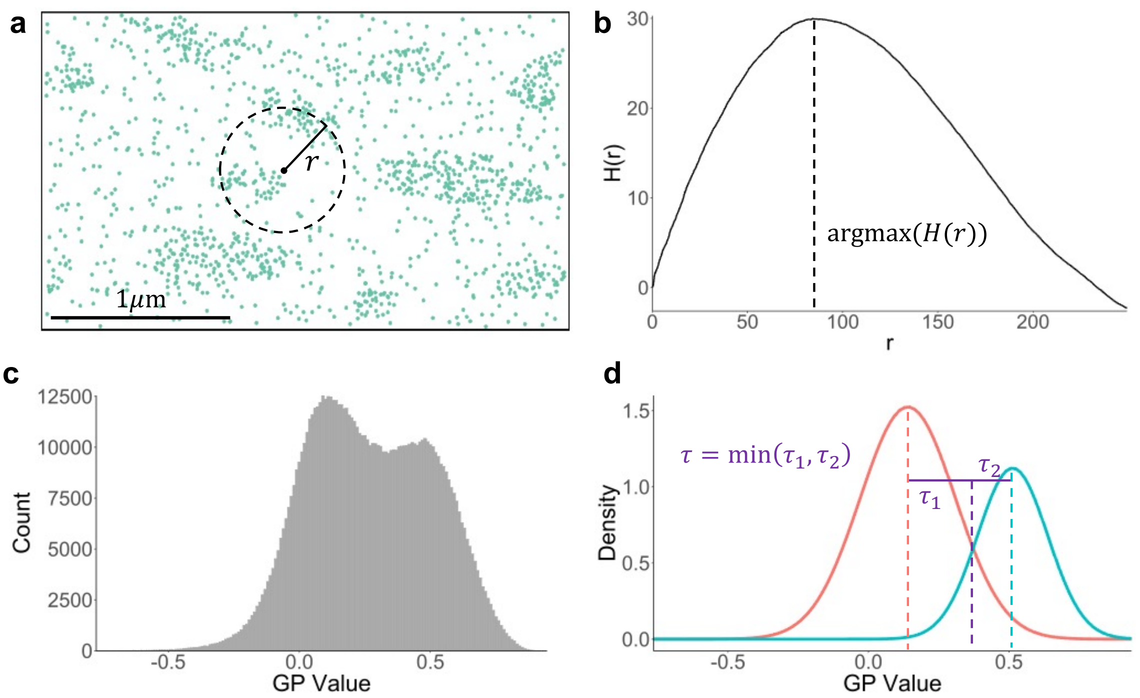

Supplementary Figure 2: **Techniques for parameter estimation**. **a)** A clustered point pattern. **b)** The Ripley's H function depicts the spatially-averaged density of points at varying radii for the point pattern in **a)**. The maximising value yields the most likely cluster radius. **c)** Histogram of the distribution of all GP values acquired from artificial membrane data. **d)** Results of fitting two Gaussian distributions to the histogram. The deviance τ is taken to be the minimum distance from either peak to the point of intersection.

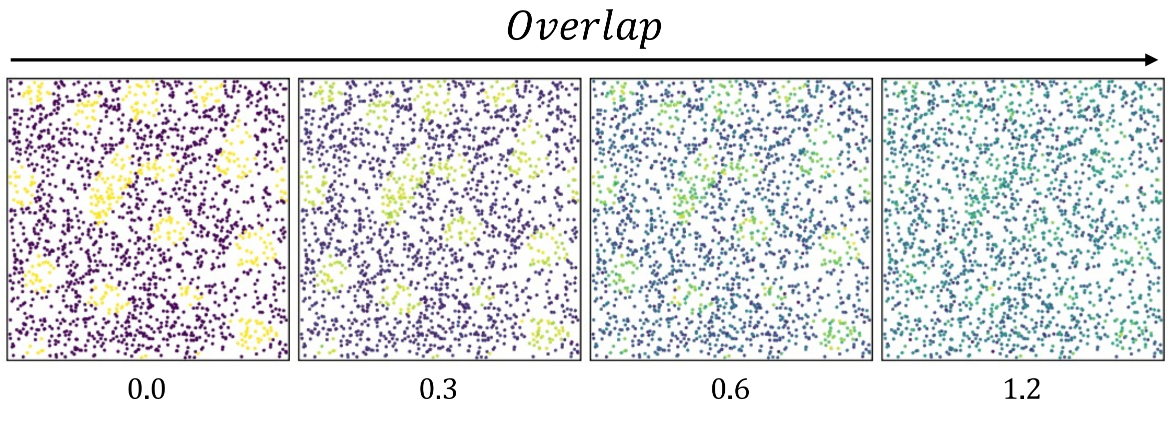

Supplementary Figure 3. A simulated point pattern with an increasing degree of overlap applied between the two normal distributions mark value. Domains become more difficult to distinguish as the overlap increases.

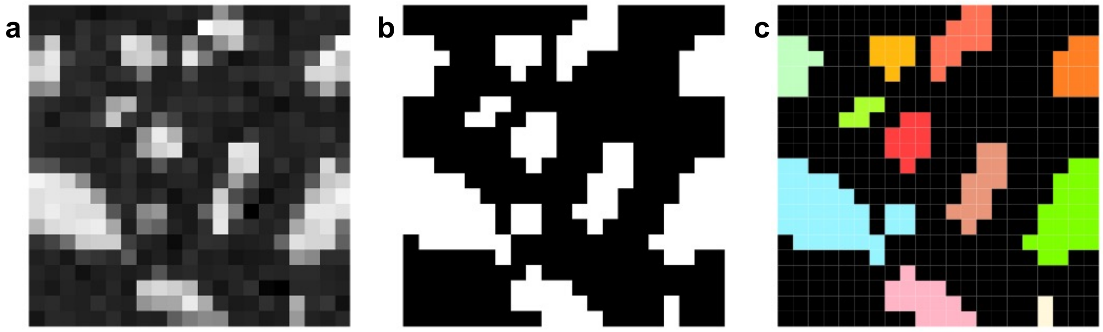

Supplementary Figure 4. Simple segmentation of high ordered domains using MOM. **a)** A grayscale variant of the MOM from **Figure 3f. b)** Simple thresholding can highlight underlying domains. **c)** Image segmentation techniques isolate individual domains, from which underlying properties can be derived per domain.

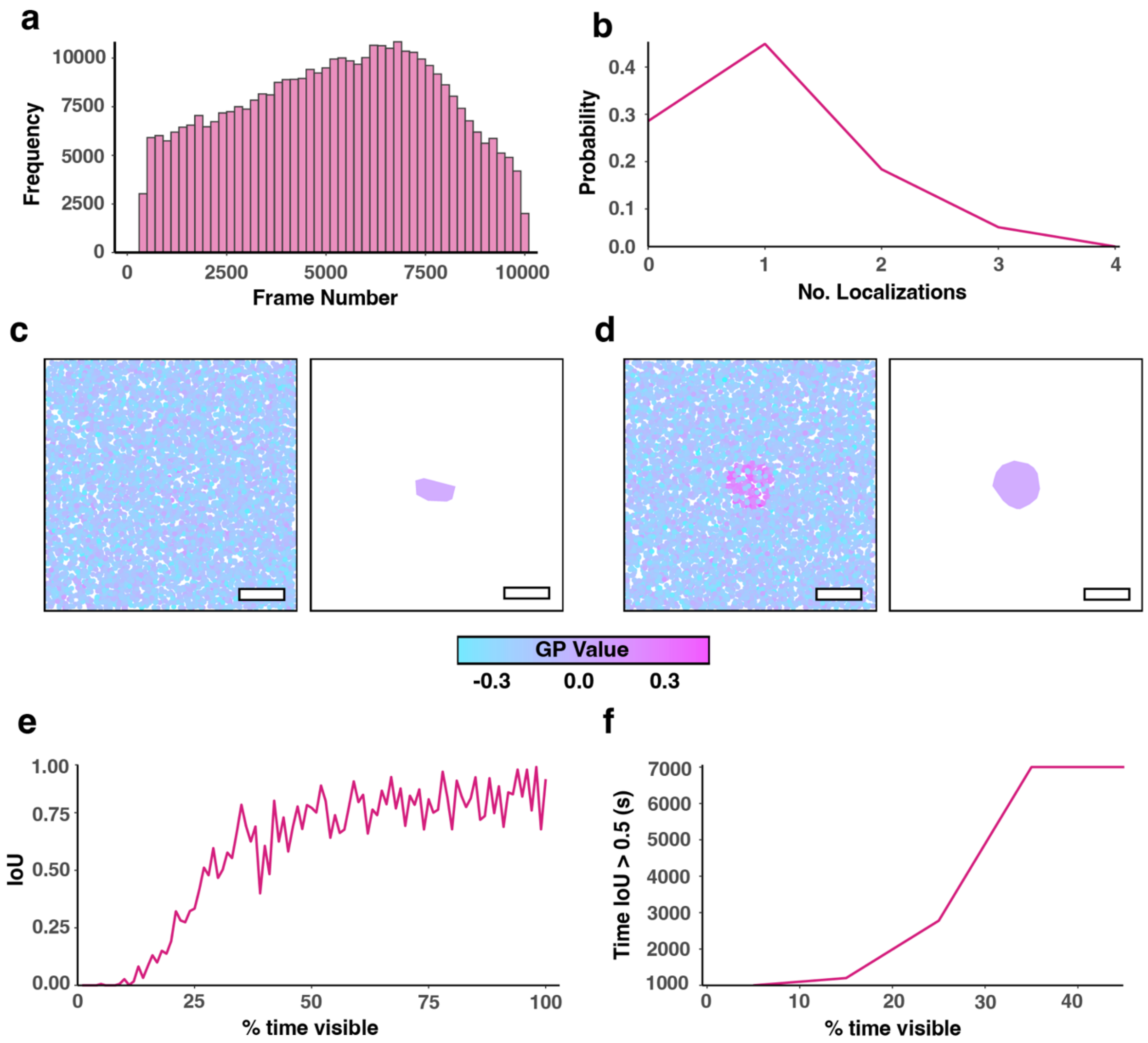

Supplementary Figure 5: Impact of domain lifetime on JOSEPH segmentation. a) A combined histogram of localizations per frame across all cell surface ROIs and **b)** the discrete probability distribution of the number of localizations in an arbitrary frame (7000). These distributions were used to determine the parameters for simulating frames of a typical acquisition, thus controlling the number of localizations per frame

for a simulated ROI. **c)** Example of point data for a simulated high order domain where the domain is present, i.e., localizations within the domain exhibit increased GP, for 20% of the acquisition (left), with the corresponding convex hull generated from JOSEPH (right). **d)** Example of point data for a simulated high order domain where the domain is present, i.e., localizations within the domain exhibit increased GP, for 80% of the acquisition (left), with the corresponding convex hull generated from JOSEPH (right). All points and hulls are psuedocoloured according to GP values. **e)** The intersection over union (i.e., the quotient of the area of the segmented domain over the area of the ground truth, IoU) score in response to simulated domain lifetime. **f)** The persistence time of high-quality JOSEPH segmentation (determined here as an IoU score greater than 0.5), in response to domain lifetime. Scale bars in all images are 500 nm.

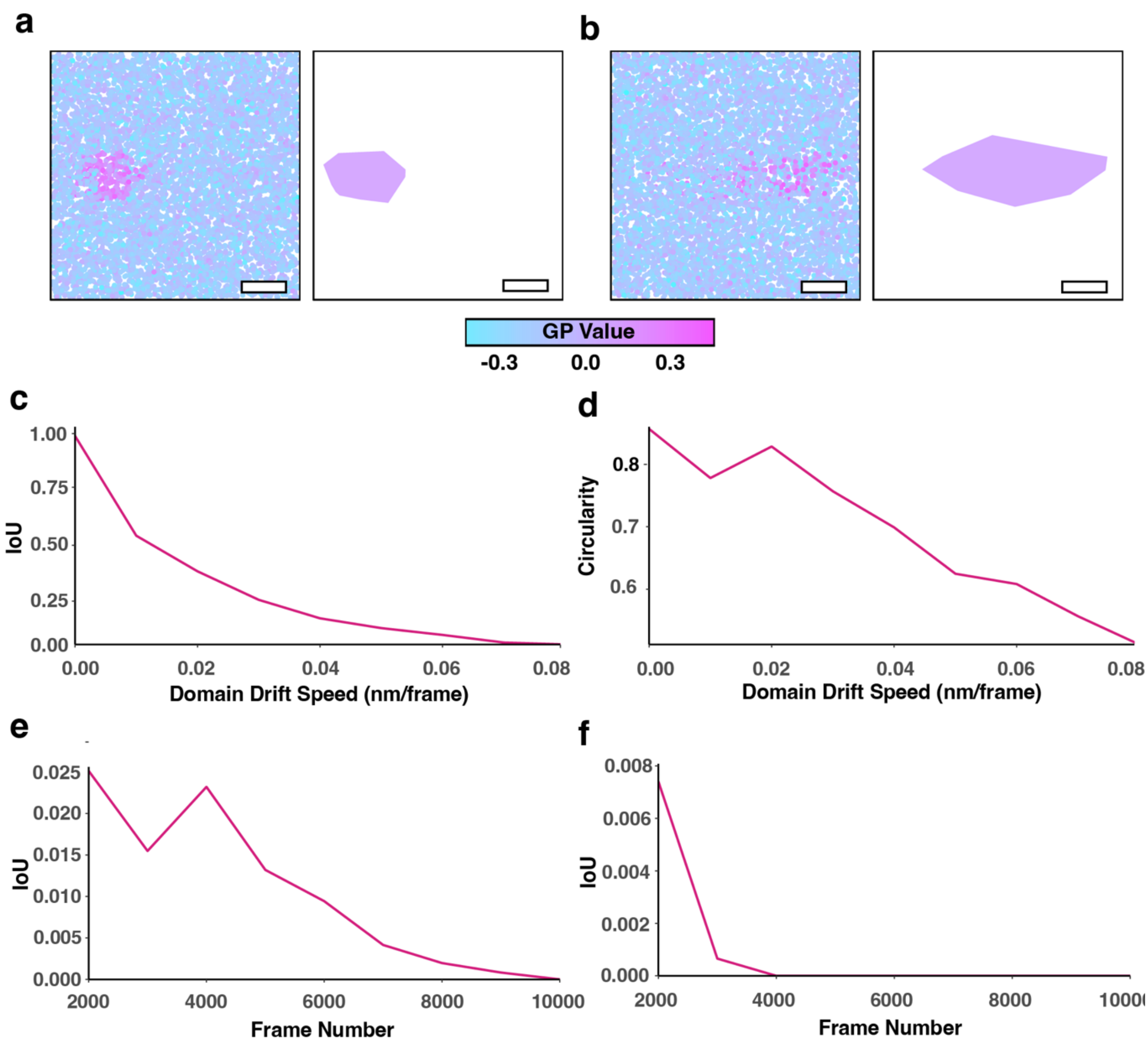

Supplementary Figure 6: Impact of domain movement on JOSEPH segmentation. **a)** Example of point data for a simulated high order domain where the domain moves at a speed of 0.02 nm/frame during the acquisition (akin to a 200 nm translocation) (left), with the corresponding convex hull generated from JOSEPH (right). **b)** Example

of point data for a simulated high order domain where the domain moves at a speed of 0.08 nm/frame during the acquisition (akin to an 800 nm translocation) (left), with the corresponding convex hull generated from JOSEPH (right). All points and hulls are psuedocoloured according to GP values. **c)** The intersection over union (i.e., the quotient of the area of the segmented domain over the area of the ground truth, IoU) score in response to domain movement speed. **d)** The final observed circularity ($4\pi A/P^2$, where A is hull area and P is perimeter) in response to domain movement speed. **e)** IoU score for hulls found in all consecutive 2000-frame time intervals, compared with the first 2000-frame interval for a drift speed of 0.02 nm/frame. **f)** IoU score for hulls found in all consecutive 2000-frame time intervals, compared with the first 2000-frame interval for a drift speed of 0.08 nm/frame. Scale bars in all images are 500 nm.

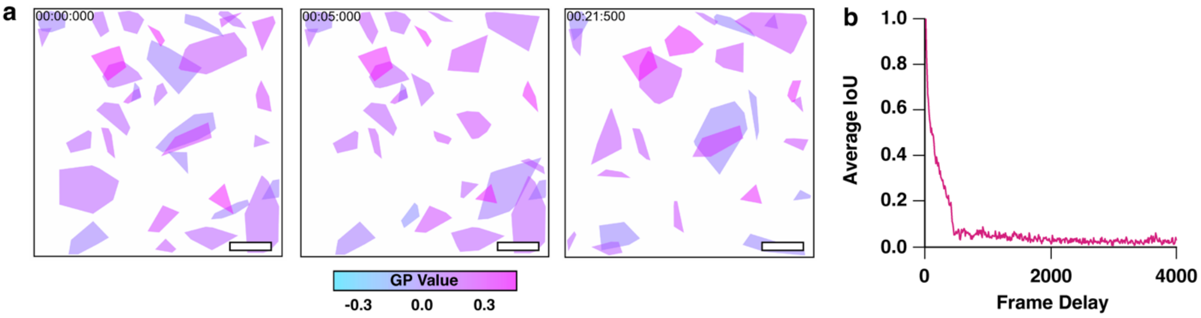

Supplementary Figure 7: Determination of IoU for time sub-sampled live cell di-4-ANNEPDHQ PAINT data. a) Exemplar JOSEPH segmentation of three separate time windows (0.0 s, 5.0 s and 21.5 s) from a single ROI from the live PAINT data. The sliding window starts at frame 0 and is 2000 frames in length, and then this window is moved by 10 frames (equivalent to 5 seconds), for each new window. All domains/hulls are psuedocoloured according to GP values. Scale bars in all images are 500 nm. **b)** The average intersection over union (IoU) score at different frame delay times., i.e., the quotient of the area of the segmented domains in the first 2000 frame block over the area of domains found at each subsequent 10-frame shifted block score at different frame delay times.

Supplementary References

1 Ripley, B. D. The Second-Order Analysis of Stationary Point Processes. *Journal of Applied Probability* **13**, 255-266, doi:10.2307/3212829 (1976).

2 Kiskowski, M., Hancock, J. & Kenworthy, A. On the Use of Ripley's K-Function and Its Derivatives to Analyze Domain Size. *Biophysical journal* **97**, 1095-1103, doi:10.1016/j.bpj.2009.05.039 (2009).

3 Scrucca, L., Fop, M., Murphy, T. B. & Raftery, A. E. mclust 5: Clustering, Classification and Density Estimation Using Gaussian Finite Mixture Models. *R J* **8**, 289-317 (2016).
