# Supplementary material for: Mapping membrane biophysical nano-environments
Source: Nat Commun. 2024 Nov 7;15:9641. doi: 10.1038/s41467-024-53883-1 (PMC11544141; doi:10.1038/s41467-024-53883-1)
Supplement: Supplementary file 4 — Reporting Summary [file 41467_2024_53883_MOESM4_ESM.pdf]

nature portfolio

Corresponding author(s): Daniel J Nieves

Last updated by author(s): Sep 23, 2024

Reporting Summary

Nature Portfolio wishes to improve the reproducibility of the work that we publish. This form provides structure for consistency and transparency in reporting. For further information on Nature Portfolio policies, see our Editorial Policies and the Editorial Policy Checklist.

Statistics

For all statistical analyses, confirm that the following items are present in the figure legend, table legend, main text, or Methods section.

n/a	Confirmed	
☐	☒	The exact sample size (*n*) for each experimental group/condition, given as a discrete number and unit of measurement
☐	☒	A statement on whether measurements were taken from distinct samples or whether the same sample was measured repeatedly
☐	☒	The statistical test(s) used AND whether they are one- or two-sided<br>*Only common tests should be described solely by name; describe more complex techniques in the Methods section.*
☒	☐	A description of all covariates tested
☒	☐	A description of any assumptions or corrections, such as tests of normality and adjustment for multiple comparisons
☐	☒	A full description of the statistical parameters including central tendency (e.g. means) or other basic estimates (e.g. regression coefficient) AND variation (e.g. standard deviation) or associated estimates of uncertainty (e.g. confidence intervals)
☐	☒	For null hypothesis testing, the test statistic (e.g. *F*, *t*, *r*) with confidence intervals, effect sizes, degrees of freedom and *P* value noted<br>*Give P values as exact values whenever suitable.*
☒	☐	For Bayesian analysis, information on the choice of priors and Markov chain Monte Carlo settings
☒	☐	For hierarchical and complex designs, identification of the appropriate level for tests and full reporting of outcomes
☒	☐	Estimates of effect sizes (e.g. Cohen's *d*, Pearson's *r*), indicating how they were calculated

Our web collection on statistics for biologists contains articles on many of the points above.

Software and code

Policy information about availability of computer code

Data collection	Simulated data sets used in this work are available at https://github.com/lucapanconi/PLASMA. The data that support the findings of this study are available from the corresponding author upon reasonable request
Data analysis	The PLASMA software package v1.0.0 was written in the R programming language v4.2.2, and employed in the integrated development environment RStudio, version 2022.07.1+558. Software is freely available under GNU General Public License v3.0 (https://github.com/lucapanconi/PLASMA).

For manuscripts utilizing custom algorithms or software that are central to the research but not yet described in published literature, software must be made available to editors and reviewers. We strongly encourage code deposition in a community repository (e.g. GitHub). See the Nature Portfolio guidelines for submitting code & software for further information.

Data

Policy information about availability of data

All manuscripts must include a data availability statement. This statement should provide the following information, where applicable:

- Accession codes, unique identifiers, or web links for publicly available datasets
- A description of any restrictions on data availability
- For clinical datasets or third party data, please ensure that the statement adheres to our policy

Simulated data sets used in this work are available at https://github.com/lucapanconi/PLASMA. The data that support the findings of this study are available from the corresponding author upon reasonable request

Research involving human participants, their data, or biological material

Policy information about studies with human participants or human data. See also policy information about sex, gender (identity/presentation), and sexual orientation and race, ethnicity and racism.

Reporting on sex and gender	N/A
Reporting on race, ethnicity, or other socially relevant groupings	N/A
Population characteristics	N/A
Recruitment	N/A
Ethics oversight	N/A

Note that full information on the approval of the study protocol must also be provided in the manuscript.

Field-specific reporting

Please select the one below that is the best fit for your research. If you are not sure, read the appropriate sections before making your selection.

☒ Life sciences ☐ Behavioural & social sciences ☐ Ecological, evolutionary & environmental sciences

For a reference copy of the document with all sections, see nature.com/documents/nr-reporting-summary-flat.pdf

Life sciences study design

All studies must disclose on these points even when the disclosure is negative.

Sample size	3900 ROIs for simulated data analysis (2000 for PLASMA, 1000 domain lifetime, 900 domain drift)<br>GUV data were taken from two independent experiments and 179 ROIS (DOPC: 93; DPPC: 86) were taken for analysis.<br>109 ROIs from two independent experiments and 6 cells total for the live cell analysis (57 untreated, and 52 methyl-beta-cyclodextrin treated)
Data exclusions	N/A
Replication	For GUV and live cell work technical replicate measurements within the same sample were taken, and the measurements reproduced.
Randomization	N/A
Blinding	N/A

Reporting for specific materials, systems and methods

We require information from authors about some types of materials, experimental systems and methods used in many studies. Here, indicate whether each material, system or method listed is relevant to your study. If you are not sure if a list item applies to your research, read the appropriate section before selecting a response.

Materials & experimental systems

n/a	Involved in the study
☒	☐ Antibodies
☐	☒ Eukaryotic cell lines
☒	☐ Palaeontology and archaeology
☒	☐ Animals and other organisms
☒	☐ Clinical data
☒	☐ Dual use research of concern
☒	☐ Plants

Methods

n/a	Involved in the study
☒	☐ ChIP-seq
☒	☐ Flow cytometry
☒	☐ MRI-based neuroimaging

Eukaryotic cell lines

Policy information about cell lines and Sex and Gender in Research

Cell line source(s)	The Rat (female) Mammary Fibroblast cell line used here was first isolated in Rudland, P. S., Twiston Davies, A. C. & Tsao, S. W. Rat mammary preadipocytes in culture produce a trophic agent for mammary epithelia-prostaglandin E2. J Cell Physiol 120, 364-376, doi:10.1002/jcp.1041200315 (1984).
Authentication	not authenticated
Mycoplasma contamination	mycoplasma negative
Commonly misidentified lines (See ICLAC register)	*Name any commonly misidentified cell lines used in the study and provide a rationale for their use.*
