# Supplementary material for: Mapping membrane biophysical nano-environments
Source: Nat Commun. 2024 Nov 7;15:9641. doi: 10.1038/s41467-024-53883-1 (PMC11544141; doi:10.1038/s41467-024-53883-1)
Supplement: Supplementary file 5 — Description of Additional Supplementary Files [file 41467_2024_53883_MOESM5_ESM.pdf]

Supplementary Movie 1:
Exemplar di-4-ANEPPDHQ ratiometric PAINT acquisition on live RAMA27 cells. **A** and **B:** Cells alone without any free di-4-ANEPPDHQ in solution, thus showing the cellular background. **C** and **D:** Show a typical region of the di-4-ANEPPDHQ PAINT acquisition on live cells where single dye binding events are observed. Left panels (**A** and **C**) show the short wavelength channel (*i.e.*, Ch1: 505-600 nm) and the right panels (**B** and **D**) show the corresponding long wavelength channel (Ch2:>640 nm). The grayscale lookup table has been inverted and rescaled to aid identification viewing of the raw data within similar colour ranges.

Supplementary Movie 2:
Exemplar JOSEPH segmentation of a complete 10 frame sliding window of a single ROI from the live PAINT data. The sliding window starts at frame 0 and is 2000 frames in length, and then this window is moved by 10 frames (equivalent to 0.5 s), for each new window (left) with areas of identified domains shown (right). All domains/hulls are psuedocoloured according to GP values. Scale bars in all images are 500 nm. For each window the area histogram of the domains segmented are shown (right).
